# Evolutionary rate variation among genes involved in galactomannan biosynthesis in *Coffea canephora*

**DOI:** 10.1002/ece3.6084

**Published:** 2020-02-11

**Authors:** Collins Ogutu, Sylvia Cherono, Charmaine Ntini, Mohammad Dulal Mollah, Lei Zhao, Mohammad A. Belal, Yuepeng Han

**Affiliations:** ^1^ CAS Key Laboratory of Plant Germplasm Enhancement and Specialty Agriculture Wuhan Botanical Garden The Innovative Academy of Seed Design Chinese Academy of Sciences Wuhan China; ^2^ Sino‐African Joint Research Center Chinese Academy of Sciences Wuhan China; ^3^ University of Chinese Academy of Sciences Beijing China

**Keywords:** *Coffea canephora*, galactomannan, nucleotide diversity, positive selection, purifying selection, synonymous and nonsynonymous substitution

## Abstract

The endosperm cell walls of mature coffee seeds accumulate large amounts of mannan storage polysaccharides, which serve as nutrient reserve for embryo and contribute to beverage quality. Our study investigated the evolutionary patterns of key galactomannan (GM) biosynthesis genes using *d*_N_/*d*_S_ ratio, synteny, and phylogenetic analysis and detected heterogeneity in rate of evolution among gene copies. Selection ratio index revealed evidence of positive selection in the branch editing gene *Coffea canephora alpha (α) galactosidase* (*Cc‐alpha Gal*) at Cc11_g15950 copy (*ω* = 1.12), whereas strong purifying selection on deleterious mutations was observed in the *Coffea canephora uridine diphosphate (UDP)‐glucose 4′‐epimerase* (*Cc‐UG4E*) and *Coffea canephora mannose‐1P guanylytransferase* (*Cc‐MGT*) genes controlling the crucial nucleotide carbon sugar building blocks flux in the pathway. Relatively low sequence diversity and strong syntenic linkages were detected in all GM pathway genes except in *Cc‐alpha Gal*, which suggests a correlation between selection pressure and nucleotide diversity or synteny analysis. In addition, phylogenetic analysis revealed independent evolution or expansion of GM pathway genes in different plant species, with no obvious inferable clustering patterns according to either gene family or congruent with evolutionary plants lineages tested due to high dynamic nature and specific biochemical cell wall modification requirements. Altogether, our study shows a significant high rate of evolutionary variation among GM pathway genes in the diploid *C. canephora* and demonstrates the inherent variation in evolution of gene copies and their potential role in understanding selection rates in a homogenously connected metabolic pathway.

## Graphical abstract

This study investigated the evolutionary patterns of key galactomannan biosynthesis genes using selection ratio index, synteny, and phylogenetic analysis and detected heterogeneity in rate of evolution among gene copies.

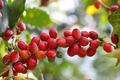

## 1 INTRODUCTION

Evolutionary studies have shown that plant genomes tend to have high rate of evolution as compared with animals due to polyploidization, local duplication events, and high retention rates of duplicate genes (Jeffrey, 2002). Gene duplication enables duplicated members to evolve under varied selective constraint (Panchy, Lehti‐Shiu, & Shiu, 2016), leading to evolutionary rate variation among copies as copies tend to undergo different selection pressure due to their contribution to fitness (Kimura, 1977; Rausher, Lu, & Meyer, 2008). Homogenously connected metabolic pathways are increasingly being used to clarify selection and evolutionary rate dynamics because genes operate as connected components based on their position, flux control, and downstream effects within the pathway (Rausher, Miller, & Tiffin, 1999). Pathway architecture has been shown to affect protein evolutionary rates (Greenberg, Stockwell, & Clark, 2008), for example, increased *d*_N_ for downstream enzymes have previously been reported in carotenoid, gibberellin, and anthocyanin pathway genes (Livingstone & Anderson, 2009; Rausher et al., 1999; Yang, Zhang, & Ge, 2009). Thus, metabolic networks create the potential for predicting differential evolutionary rates as a function of the network properties. For example, low evolutionary rates in upstream genes have been reported due to their flux control properties (Vitkup, Kharchenko, & Wagner, 2006). In addition to pathway properties, rapid evolutionary rate pattern has been reported in regulatory genes than in structural genes (Purugganan & Wessler, 1994; Tucker & Lundrigan 1993), and in some plant lineages as a result of environmental factors (Pierce & Crawford, 1997).

Cell wall storage polysaccharides (CWSPs) are mainly composed of mannans, glucomannans, galactomannans, xyloglucans, and galactans. These polysaccharides structurally vary in backbone chain, branching residue and nucleotide sugars, and their biosynthesis requires different hydrolytic enzymes (Buckeridge & Reid, 1996). CWSPs of the mannan family are reserved in considerable amount as nutrient source for germinating embryo in endosperm of many albuminous monocot and dicot seed plants, such as date palm, guar, and coffee (Buckeridge, 2010). In addition, the mannan CWSPs enable seeds to retain living cellular endosperm at mature, confer seed firmness, and contribute to water buffering during drought (Buckeridge, 2010; Horbowicz & Obendorf, 1994). The galactomannans (GMs) structure consist of a linear mannose backbone chain with galactose side chains which vary in mannose: galactose ratio among species, for example, 1:1 and 20:1 in fenugreek and tobacco, respectively (Reid, Edwards, Dickson, Scott, & Gidley, 2003). The mannose/galactose ratio variation in legumes subfamilies has been shown to reflect systematics and evolutionary pattern in their adaptation to tropical and temperate environments. For example, tropical legumes exhibit high content of galactomannan as well as mannose/galactose ratio, which could be used as taxonomic markers (Buckeridge & Reid 1996; Polhill et al.,; Siol, Wright, & Barrett, 2010).

**Figure 1:**
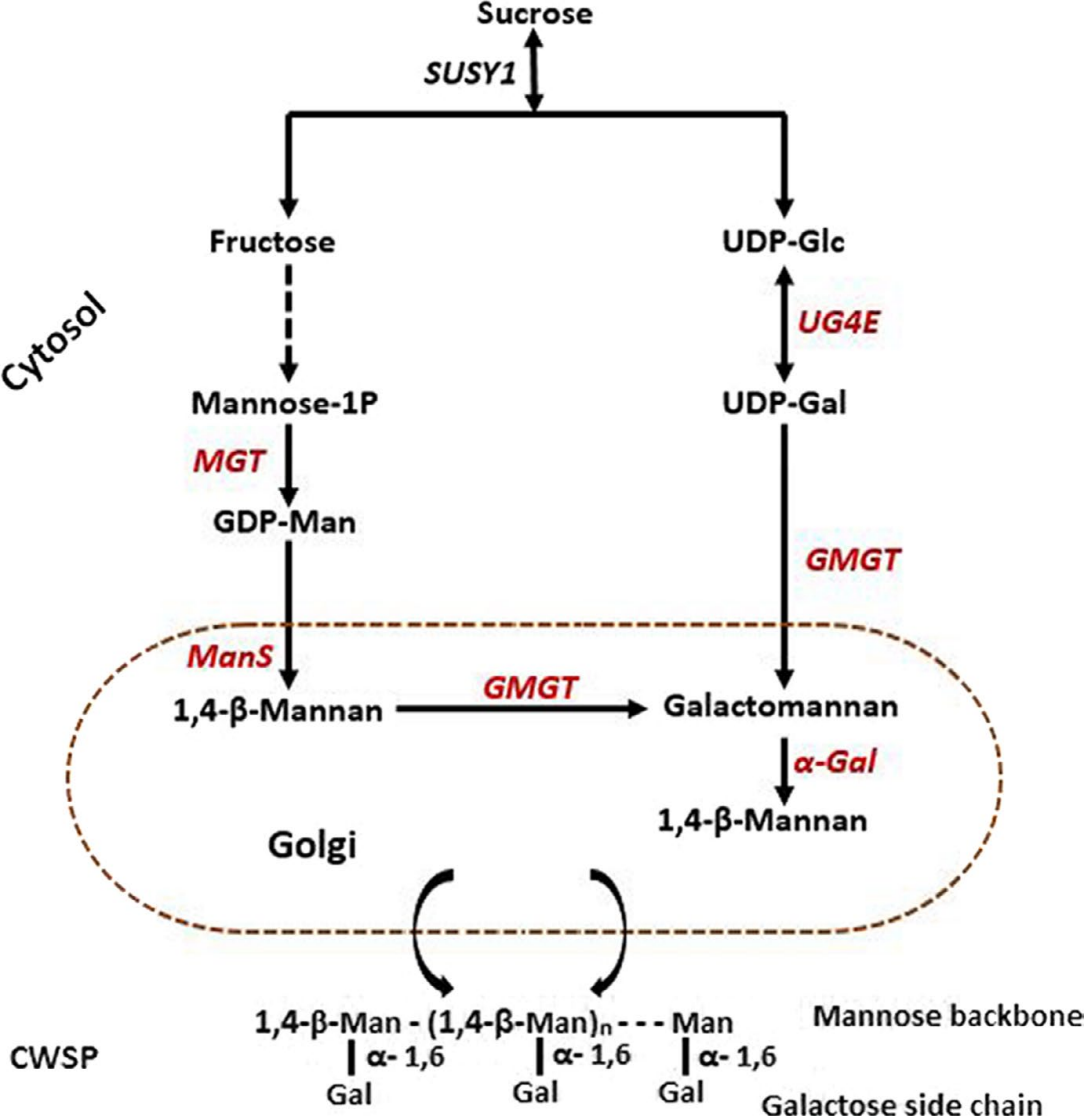
Simplified Galactomannan biosynthetic pathway in coffee seed

The endosperm of coffee seeds predominantly accumulates galactomannans, which account for over 50% of all CWSPs (Redgwell & Fischer, 2006). The biosynthetic pathway of galactomannans has been well characterized in plants (Dhugga, Barreiro, & Whitten, 2004; Edwards, Scott, Gidley, & Reid, 1992; Joersbo, Marcussen, & Brunstedt, 2001; Pré, Caillet, Sobilo, & McCarthy, 2008) and involves genes encoding mannose‐1P *guanylytransferase* (MGT), uridine diphosphate (UDP)‐glucose 4′‐epimerase (UG4E), mannan synthase (ManS), galactomannan galactosyltransferase (GMGT), and α‐galactosidase (α‐Gal) (Figure 1). MGT catalyzes the conversion of Mannose‐1P to GDP‐Mannose (GDP‐Man), which is subsequently converted to 1,4‐β‐Mannan by mannan synthase (ManS), whereas, UG4E catalyzes the conversion of UDP‐glucose to UDP‐galactose (UDP‐Gal). GMGT converts 1,4‐β‐Mannan to galactomannan through catalyzing the formation of galactose side branch from UDP‐Gal, and α‐Gal is responsible for the regulation of postdepositional substitution rate of galactose side branch.

**Figure 2:**
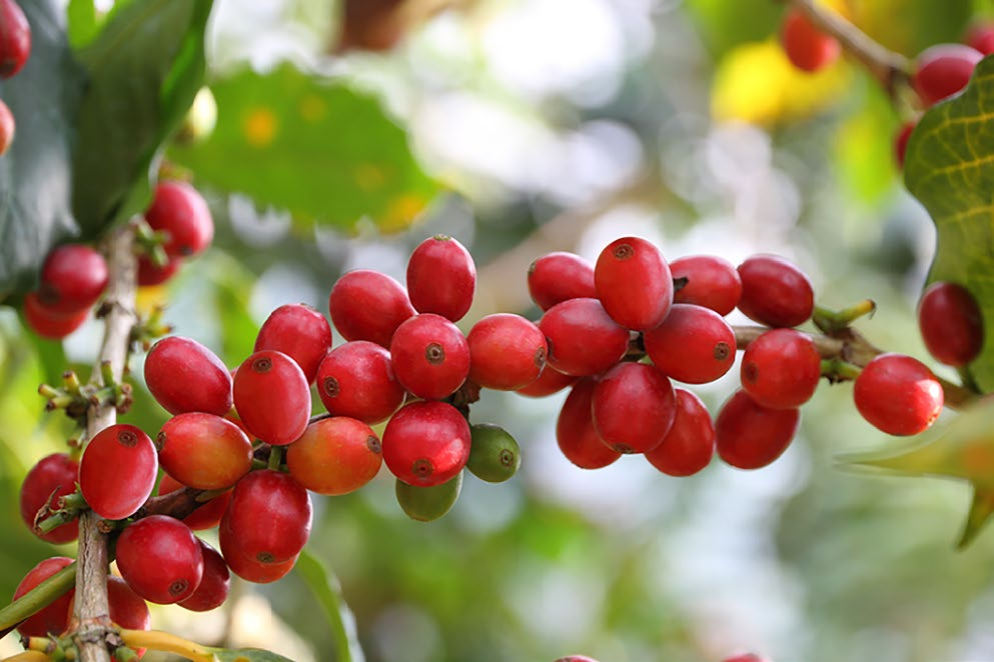
Coffee plant with mature cherries

Coffee belongs to the *Rubiaceae* family, the genus Coffea with approximately 124 species. It is a tropical plant (Figure 2) with *Coffea arabica* and *Coffea canephora* as the two widely cultivated species. The enormous coffee endosperm which constitutes approximately 99% of total seeds mass along with its ability to accumulate large amount of galactomannans makes it a suitable model plant for investigating metabolism as well as the evolution of galactomannan pathway genes. The structure of the diploid *C. canephora* genome suggests that it has not undergone whole‐genome duplication (WGD) since triplication origin from core eudicots, and gene expansion is mainly attributed to local duplication (Denoeud et al., 2014). For example, tandem duplication in caffeine metabolic pathway genes has been implicated in convergent adaptive evolution in coffee. In this study, we investigated the evolutionary patterns of galactomannan biosynthesis genes in coffee using selection pressure, synteny, and phylogenetic analysis with an aim of demonstrating variation in evolution using multiple‐copy analysis in *C. canephora*.

## 2 MATERIALS AND METHODS

### 2.1 Plant material, DNA extraction, amplification, and sequencing

A total of 23 *C. canephora* including 18 cultivated and five wild accessions used in this study are maintained at Spice and Beverage Research Institute of Chinese Academy of Tropical Agricultural Sciences (Hainan, China). Cultivated accessions were selected based on our previously published polymorphic data (Ogutu et al., 2016; Yan et al., 2019). Seeds were randomly collected at mature stage, immediately frozen in liquid nitrogen, and then stored at −80°C until use. Total DNA was isolated from twenty‐three freshly frozen coffee seeds using our previously described modified protocol (Ogutu et al., 2016). PCR primers were designed for specific sequencing regions (Table S1). Amplification was carried out in a 50 μl reaction volume on a GeneAmp PCR System 9700 (ABI), containing a 5 μl 10 Ex‐PCR buffer (100 mM Tris‐HCl pH 8.8 at 25°C; 500 MmKCl, 0.8% [v/v] Nonidet), 4 μl 10 m MdNTPs, 1.5 μl 10 μM of each primer, 0.5 μl Ex‐Taq DNA polymerase (5 U/μl) (Sangon), 3 μl sample DNA, 34.5 μl ddH_2_O. PCR program was optimized as follows: an initial denaturing for 4 min at 95°C, followed by 40 cycles of 94°C for 30 s, 52–61°C Tm for 40 s, and 72°C for 2:30 min; and a final extension at 72°C for 10 min. PCR products were sequenced directly after separation on a 1% agarose gels stained with GoldView™ (ZomanBio) and purification using TIANgel Midi Purification kit (TIANGEN Biotech). All DNA sequences have been submitted to the GenBank databases with BankIt accession numbers:‐ BankIt2304042: MN960694–MN960808; BankIt2304536: MN960809–MN960900, BankIt2304544: MN960901–MN960969, BankIt2304547: MN960970–MN961015, BankIt2304548: MN961016–MN961107.

### 2.2 Sequence retrieval and alignment

Nonredundant NCBI blast search (Gish & States, 1993) was conducted to identify coding DNA sequences of *C. canephora* galactomannan gene copies using previously reported coding DNA sequences for *Cc‐alpha*, *Cc‐Man‐S*, and *Cc‐alpha Gal* as query sequences (Joët et al., 2014; Pré et al., 2008), except for *Cc‐MGT* and *Cc‐UG4E* whose query sequences were retrieved from *Arabidopsis* and rice, respectively (Conklin et al., 1999; Guevara, El‐Kereamy, Yaish, Mei‐Bi, & Rothstein, 2014). The partial *Cc‐UG4E* and *Cc‐MGT* sequences (Joët et al., 2014) were used to blast *Arabidopsis* and rice databases and the best hits results were selected and subsequently used to retrieve complete coding sequences from *C. canephora* genome after confirming their annotation.

### 2.3 Sequence diversity and codon usage bias test

The average pairwise nucleotide sequence diversity index, *π*, was calculated using Jukes and Cantor correction method in DnaSP v5.0 (Librado & Rozas, 2009). Effective number of codon (ENC) was tested in DnaSP and confirmed in CodonW (Peden, 1999). ENC is a codon usage bias test that can reflect the level of selective constraint on a particular gene and variation in synonymous substitution rate is related to codon usage (Sharp, 1991; Sharp, Tuohy, & Mosurski, 1986). Low ENC values represent greater codon bias, while high ENC represents low codon usage bias (Wright, 1990).

### 2.4 Variation in *d*_N_, *d*_S,_ and *ω* among genes

The synonymous (*d*_S_) and nonsynonymous (*d*_N_) nucleotide substitution rates were calculated using CodeML feature in the phylogenetic analysis by maximum likelihood, PAML v4.3 (Yang, 1997) as described by (Jeffares, Tomiczek, Sojo, & Reis,). Briefly, coding DNA sequences were translated to protein sequences, and their orthology determined for tree construction and CodeML analysis in PALM. Overall, cumulative mutations across the whole gene region can effectively be used to estimate positive selection or selective pressure by calculating substitution ratio of nonsynonymous and synonymous mutations (*d*_N_/*d*_S_ = *ω*). Under neutral selection, both nonsynonymous and synonymous are fixed at the same rate thus *ω* = 1. Purifying selection prevents fixation of deleterious mutation, thus *ω* < 1, due to excess synonymous over nonsynonymous substitutions. When nonsynonymous mutation offers selective advantage, is it fixed at a higher rate than synonymous mutation? Thus, *ω* > 1 (Yang & Nielsen, 2000). Due to complex demographic history of domesticated plants, parameters allowing for deviations from the standard neutral model were used to best reflect specific demographic events (Wright & Gaut, 2005). Evidence of positive selection was determined by calculating selection at each codon for all genes using likelihood‐ratio test (LRT) to compare M8 and M8a models (Swanson, Nielsen, & Yang, 2003), which allow for positive selection (beta, and *ω* > 1) and near neutral model M8a (*ω* = 1), respectively, for gene copies in PAML. Branch‐site analysis with model A was used to detect independent *ω* values of positive selection, and Bayes Empirical Bayes analysis (BEB) was performed to identify sequences under selection. For statistical significance, bivariate correlation between sequence length and mutation rates *d*_S_, *d*_N,_ and *ω* was tested to confirm reliability of variation in the data.

### 2.5 Phylogenetic and synteny analysis of galactomannan pathway genes

All‐against‐all BlastP in different plant genome databases was conducted during the period of March 2018 to retrieve putative galactomannan pathway gene copies and their corresponding annotations using default parameters (Altschul, Madden, Schaffer, Zhang, & Zhang, 1997; Gu, Cavalcanti, Chen, Bouman, & Li, 2002). Homology was inferred for sequences with at least 40% identity and 70% align‐able region lengths and subsequently used for phylogenetic analysis. Protein sequence alignment was performed using ClustalW v1.83 (Thompson, Higgins, & Gibson, 1994) with the default parameters and corrected manually on MEGA v.6.0 (Tamura, Stecher, Peterson, Filipski, & Kumar, 2013). Prior to ClustalW alignment, redundant sequences were filtered using CD‐HIT program (Huang, Niu, Gao, Fu, & Li, 2010). Conserved sequences were trimmed and then used to guide CDS alignments in BioEdit 7.0.5.3 (Hall, 1999). Tree construction was conducted using bootstrap neighbor‐joining method in MEGA v6.0, with algae as outgroup, a Kimura 2‐parameters, and a bootstrap value of 1,000 to assess internal node stability.

Conserved synteny analysis of short chromosomal segments that included regions of GM pathway genes was conducted to visualize syntenic linkages in the *C. canephora* genome using genome comparison visualizer in Easyfig software (Sullivan, Petty, & Beatson, 2011). Alignment of chromosome segments that contained putative GM pathway gene copies and their flanking regions extending 100 kb in both forward and reverse was conducted in MAFFTv6.7 (Kazutaka & Daron, 2013), and two chromosome regions with best alignment hits were selected for synteny analysis.

## 3 RESULTS

### 3.1 Analysis of sequence polymorphism and codon usage bias

A total of 18 GM pathway gene copies were identified in *C. canephora* (*Cc*) genome and used for sequence variation test and evolutionary rates analysis. Evolutionary rate at silent (*d*_S_) and replacement (*d*_N_) mutation, nucleotide sequence polymorphism, and *ω* was compared among multiple gene copies of the GM pathway (Table 1). Overall, the average nucleotide sequence diversity (*π*) ranged from 0.03 in *Cc‐MGT* gene copies to 1.13 in *Cc‐alpha Gal* genes. Polymorphic variation within each gene copies varied from 0.02 to 0.24 for Cc08_g06540 and Cc10_g07720 in *Cc‐ManS*; from 0 to 0.18 for Cc07_g07210 and Cc07_g07220 in *Cc‐GMGT* and from 0.08 to 0.74 for Cc11_g00330 and Cc11_g15950 in *Cc‐alpha Gal* genes copies (Table 1). In contrast, a relatively low sequence polymorphism was observed in *Cc‐UG4E* and *Cc‐MGT* gene copies. Most codon usage bias was detected among *Cc‐UG4E* and *Cc‐MGT* gene copies based on lowest average ENC of 38.9 and 40.9, respectively, while *Cc‐alpha Gal* gene with the highest average *d*_N_/*d*_S_ showed least codon usage bias with a mean average ENC of 57.8 (Table 2).

**Table 1:** Sequence diversity and selection test in 23 sequenced genotypes

Enzyme	Gene locus	*π*	*d*_S_	*d*_N_	*d*_N_ */d*_S_ (*ω*)	LRT
*Cc‐Man‐S*	Cc06_g04240	0.09	0.14	0.02	0.14	0.00
	Cc01_g05870	0.47	0.31	0.11	0.35	0.00
	Cc06_g05120	0.19	0.03	0.01	0.33	1.23^NS^
	Cc08_g06540	0.02	0.06	0.00	0.00	0.00
	Cc10_g07720	0.24	0.98	0.72	0.73	0.00
*Cc‐GMGT*	Cc07_g07220	0.18	0.28	0.11	0.39	0.00
	Cc07_g07210	0.00	0.00	0.00	0.00	0.00
	Cc00_g25840	0.01	0.00	0.00	0.00	0.00
	Cc03_g01930	0.20	0.21	0.03	0.14	24.51
*Cc‐alpha Gal*	Cc02_g05490	0.16	0.41	0.29	0.71	7.94^NS^
	Cc11_g15950	0.74	0.14	0.17	1.21	98.83
	Cc04_g14280	0.15	1.07	0.58	0.54	0.00
	Cc11_g00330	0.08	0.40	0.29	0.73	48.12
*Cc‐UG4E*	Cc11_g04810	0.04	0.34	0.02	0.06	0.00
	Cc07_g03170	0.02	0.24	0.03	0.13	0.00
	Cc00_g06000	0.14	0.31	0.01	0.03	0.00
*Cc‐MGT*	Cc05_g00320	0.02	0.04	0.00	0.00	0.00
	Cc02_g07370	0.01	0.05	0.02	0.25	0.00

Note

*p* < .001.

Abbreviations: *d*_N_, nonsynonymous substitution; *d*_S_, synonymous substitution; LRT, likelihood‐ratio test; NS, not significant (*p* < .51); *π*, nucleotide sequence diversity index; *ω*, selection index.

**Table 2:** Information of the *C. canephora* GM genes used in this study

Enzyme	Gene locus	Chr. Location	Length sequenced (bp)			Coding region	
			Total	CDS	Promoter	ENC	GC (GC_3_)^a^
*Cc‐Man‐S*	Cc06_g04240	6	2,274	1,593	681	41.24	0.36 (0.41)
	Cc01_g05870	1	2,245	2,031	214	52.51	0.39 (0.43)
	Cc06_g05120	6	2,076	1,602	474	39.11	0.35 (0.31)
	Cc08_g06540	8	2,117	2,082	35	38.95	0.37 (0.30)
	Cc10_g07720	10	2,127	2,062	65	67.21	0.39 (0.32)
*Cc‐GMGT*	Cc07_g07220	7	2,233	1,335	898	54.56	0.34 (0.40)
	Cc07_g07210	7	2,218	1,347	871	37.71	0.28 (0.32)
	Cc00_g25840	0	2,120	1,311	809	39.93	0.30 (0.39)
	Cc03_g01930	3	1,823	1,383	440	50.13	0.31 (0.34)
*Cc‐alpha Gal*	Cc02_g05490	2	2,157	1,275	882	58.24	0.41 (0.48)
	Cc11_g15950	11	2,118	1,182	936	54.79	0.53 (0.52)
	Cc04_g14280	4	2,130	1,137	993	61.01	0.41 (0.37)
	Cc11_g00330	11	1,754	1,311	443	57.20	0.41 (0.49)
*Cc‐UG4E*	Cc11_g04810	11	2,142	1,355	787	40.91	0.48 (0.57)
	Cc07_g03170	7	2,177	2,116	61	43.42	0.40 (0.53)
	Cc00_g06000	0	1,928	1,350	578	38.33	0.38 (0.36)
*Cc‐MGT*	Cc05_g00320	5	2,128	1,086	1,042	36.47	0.44 (0.58)
	Cc02_g07370	2	1,736	1,086	650	41.31	0.37 (0.43)

Abbreviation: ENC, effective number of codon.

a Average for 23 *C. canephora* accessions, GC_3_, average GC content at the third‐codon positions of coding genes for all accessions

### 3.2 Comparing *d*_N_, *d*_S,_ and *ω* among gene copies

**Figure 3:**
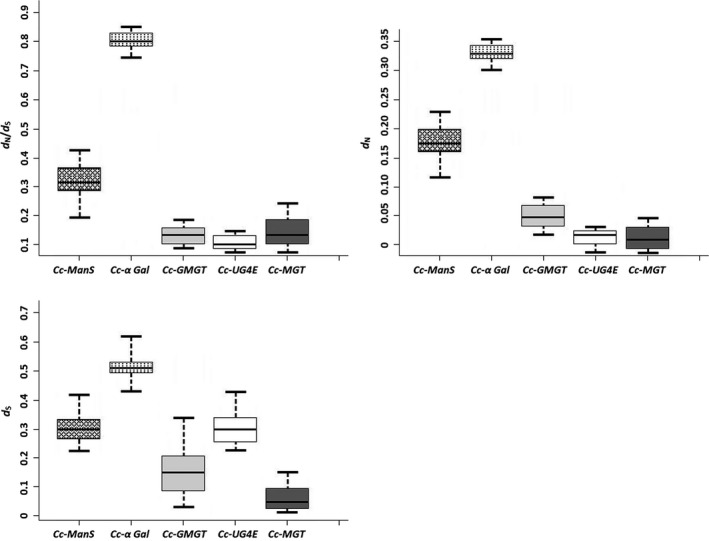
Evolutionary rates at silent and replacement sites of the GM pathway genes. Average values of *d*_S_, *d*_N,_ and *d*_N_/*d*_S_ are represented by horizontal bars within the boxes at 95% confidence intervals

The average *d*_S_ and *d*_N_ values are shown in Figure 3. Interestingly, variation pattern suggesting a slow evolutionary rate for the most upstream pathway genes as compared with the most downstream genes was observed. For example, low average *d*_S_ and *d*_N_ values were observed for two upstream genes, *Cc‐UG4E* (*d*_N_ = 0.02; *d*_S_ = 0.30; *p* < .001) and *Cc‐MGT* (*d*_N_ = 0.01; *d*_S_ = 0.05; *p* < .001), while significantly higher average *d*_S_ and *d*_N_ values were detected for the downstream *Cc‐alpha Gal* gene (*d*_N_ = 0.33; *d*_S_ = 0.51; *p* < .001) (Figure 3; Table 1). The highest *d*_S_ and *d*_N_ values were observed in *Cc‐alpha Gal* at Cc04_g14280 (*d*_S_ = 1.07) and in *Cc‐Man‐S* at Cc10_g07720 (*d*_N_ = 0.72), respectively. Test for positive selection using *d*_N_/*d*_S_ variation among gene copies revealed an excess of replacement substitution in *Cc‐alpha Gal* at Cc11_g15950 (Table 1). This result was confirmed by maximum likelihood test of *ω* in PAML, which revealed strong evidence of significant positive selection sites based on LRT values in *Cc‐alpha Gal* gene at Cc11_g15950; Cc11_g00330 with LRT of 98.83 (*p* < .001); 48.12 (*p* < .001), respectively, and in *Cc‐GMGT* gene at Cc03_g01930 with LRT of 24.51 (*p* < .001). It is noteworthy that high LRT of 1.23 and 7.94 for Cc‐ManS at locus Cc06_g05120 and Cc‐alpha Gal at locus Cc02_g05490, respectively, were observed. However, the values were not significant which could be due to the effects of either sequence length, sequence divergence, and the strength of positive selection which have been shown to affect the power of LRT (Maria, Joseph, & Ziheng, 2001).

In addition, increased nonsynonymous substitution was detected in *Cc‐Man‐S* gene at Cc10_g07720 (*ω* = 0.73) and in *Cc‐alpha Gal* gene at Cc02_g05490 (*ω* = 0.71), Cc04_g14280 (*ω* = 0.54) and Cc11_g00330 (*ω* = 0.73). However, the LRT test was not significant for Cc02_g05490. Overall, high *d*_N_/*d*_S_ ratio variation among and within gene copies was observed, which demonstrates a significantly diverse evolutionary rate in GM pathway genes. These results suggested different rates of selection events on GM pathway gene copies in *C. canephora*. More selection events appeared to have occurred in *Cc‐alpha Gal* gene, while *Cc‐UG4E* and *Cc‐MGT* copies showed evidence of strong selective constraint. However, interpreting whether the observed difference in selection rates across the pathway is actually more than expected at random requires conjunction of this data with results from population genetics models that include recombination between selected sites and nearby neutral marker (Charlesworth, 2006; Schmid, Ramos‐Onsins, Ringys‐Beckstein, Weisshaar, & Mitchell‐Olds, 2005).

### 3.3 Synteny and phylogenetic analysis

**Figure 4:**
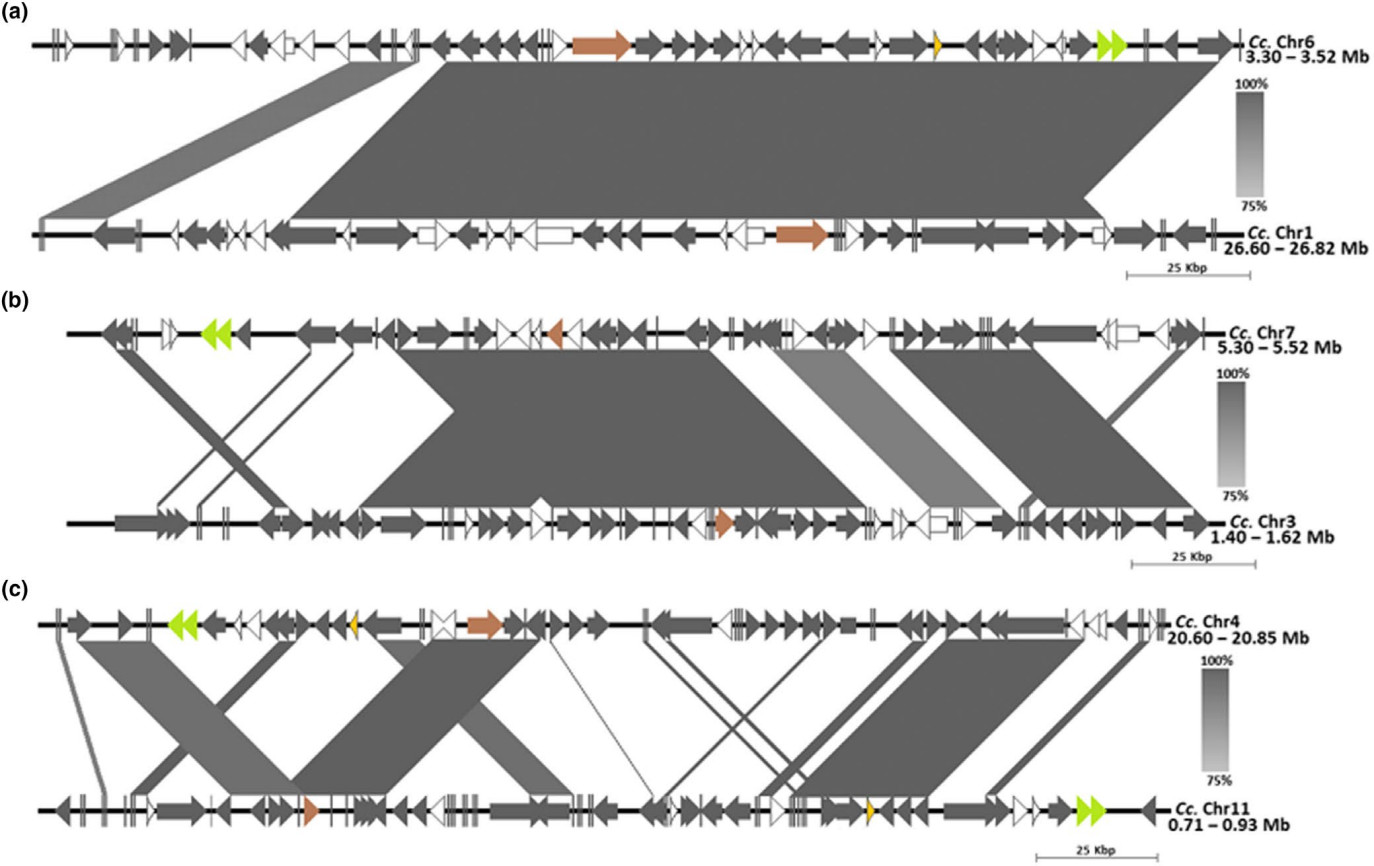
Synteny analysis of tandem chromosomal regions for *Man‐S* (a), *Cc‐GMGT* (b), showing extensive alignments, and in *Cc‐alpha Gal* (c), with less conserved (<60%) syntenic alignment of the two chromosome segments. Arrow heads represent different segment features, and target gene position is shown in dark brown color. Color intensity of gray diagonal blocks represents rate of conserved sequence sites on chromosomes

Phylogenetic and synteny analysis was conducted to get insight into evolutionary history of GM pathway gene copies. Extensive synteny was observed for most sequence segments with strong conservation of tandemly linked regions spanning ~60% in length for duplicate copies in *Cc‐Man‐S*, *Cc‐GMGT*, *Cc‐UG4E*, and *Cc‐MGT* (Figure 4; Figure S1). In contrast, a relatively weak synteny was detected at *Cc‐alpha Gal*, which might be due to more selection events accompanying its duplication in *C. canephora*.

**Figure 5:**
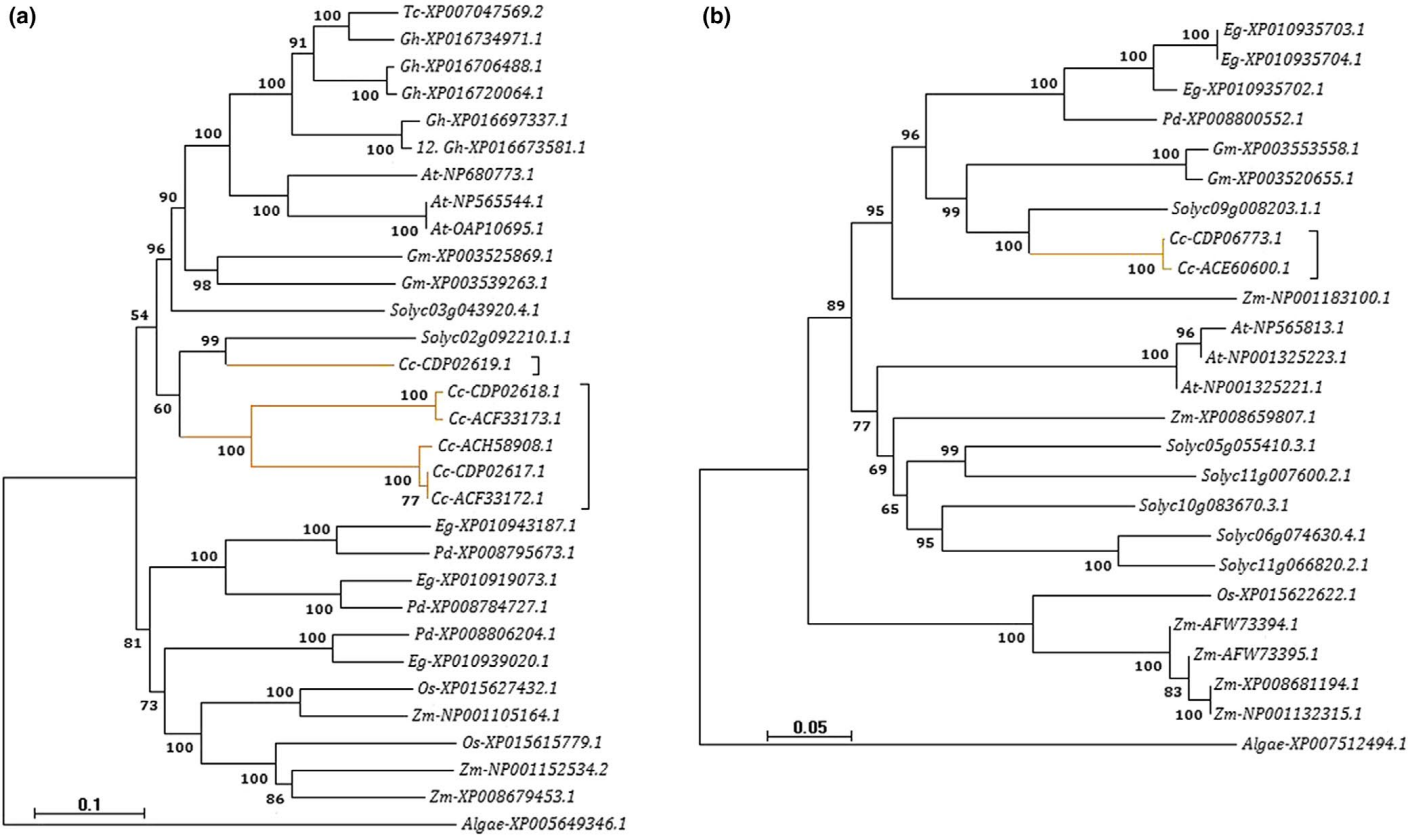
Consensus phylogenetic trees of (a) *GMGT* and (b) *Man‐S* gene family members from different plant lineages. Italicized abbreviations represents species name: *Tc*—*Theobroma cocoa*, *Gh*—*Gossypium hirsutum*, *At*—*Arabidopsis thaliana*, *Cc*—*Coffea canephora*, *Gm*—*Glycine max*, *Eg*—*Elaeis guineensis*, *Pd*—*Phoenix dactylifera*, *Os*—*Oryza sativa*, *Zm*—*Zea mays*, *Solyc*—*Solanum lycopersicum*. Gene accession numbers are shown in‐front of species name, and bootstrap values indicating confidence levels ≥50 for clustering are shown at the nodes. Tree branches for *C. canephora* copies are highlighted in brown color

The phylogenetic analysis, however, failed to support nucleotide diversity in the dataset with paralogs in most species tested forming distinct subclusters. In addition, no obvious orthologous clustering congruent with species evolution could be inferred. Each gene family appeared to have unique expansion patterns or have undergone independent evolutionary events in different plant lineages analyzed in this study. In addition, analysis of *GMGT* and *UG4E* phylogenies indicated independent subclades that corresponded with monocots and dicots groups (Figure 5; Figures S2 and S3).

## 4 DISCUSSION

Our study examined the evolutionary rate analysis of the galactomannan pathway genes based on multiple copies analysis in the diploid *C. canephora* this is because multiple‐copy encoding enzymes can provide direct evidence of gene evolution patterns in metabolic pathways (Chu, Wang, Cheng, Yang, & Yu, 2014). Five wild and eighteen accessions representing independent, divergent species (Kryazhimskiy & Plotkin, 2008; Nielsen & Yang, 2003) were sequenced to analyze evolutionary patterns in 18 GM pathway gene copies. Differential selection rates on genes cause effect on patterns of nucleotide sequence diversity (Ma et al.,^2015^, Ramsay, Rieseberg, & Ritland, 2009). In this study, we detected a correlation between different selection pressure and patterns of sequence diversity, which was consistent with the observed level of syntenic linkage analysis. The *Cc‐alpha Gal* gene showing evidence of positive and/or relaxed selection pressure had the highest sequence diversity index and displayed weak synteny. In contrast, *Cc‐UG4E* and *Cc‐MGT* under intense purifying selection had the lowest sequence diversity and displayed the strongest syntenic linkage. *Cc‐UG4E* and *Cc‐MGT* control the crucial carbon flux step from sucrose to galactomannan through synthesis of UDP‐galactose and GDP‐mannose nucleotide sugar building blocks, respectively. Thus, a strong negative selection is needed to optimize and remove deleterious mutations (Lu & Rausher, 2003) to ensure their novel evolutionary function remains fixed in the population. In addition, previous studies reveal that high gene expression is correlated to their fitness and significant role in survival (Blanc & Wolfe, 2004; Popescu, Borza, Bielawski, & Lee, 2006). Fitness genes exhibit strong codon bias due to intense purifying selection for translation efficiency and their expression levels are usually higher (Blanc & Wolfe, 2004; Brown & Kelly, 2018; Lavin, Herendeen, & Wojciechowski, 2005). More recently, expression of core GM pathway genes, including *Cc‐UG4E* and *Cc‐MGT*, was reported to be positively correlated with the amount of stored cell wall storage polysaccharides (Joët et al., 2014). Altogether, these reports are in agreement with the strong negative selection and greater codon bias observed for *Cc‐UG4E* and *Cc‐MGT* in our study.

Frequency of positive selection and selective constraints are most likely factors to account for evolutionary variation among gene copies in a metabolic pathway (Braverman, Hamilton, & Johnson, 2016; Gaut, Yang, Takuno, & Eguiarte, 2011; Yang et al., 2009). High ratio of synonymous (silent) to nonsynonymous (replacement) variation among and within gene copies observed in this study demonstrates a significant rate of evolutionary heterogeneity in GM pathway genes, which is consistent with high degree of endogenous heterogeneity reported in several cell wall storage polysaccharides (Gruppen, Kormelink, & Voragen, 1993; Toole et al., 2012). The *d*_N_/*d*_S_ ratio and codon‐based analysis detected signature of positive selection in *Cc‐alpha Gal* gene copy. Interestingly, different isoforms of *α‐Gal* gene have been reported to exist in plant tissues (Chrost & Schmitz, 2000; Dey & Del Campillo 1984) which could contribute to high rate gene mutation (Lynch & Conery, 2000). In addition, enzyme position in a metabolic pathway has been linked to evolutionary rate variation with upstream genes evolving more slowly than the downstream genes (Clotault, Peltier, Soufflet‐Freslon, Briard, & Geoffriau, 2012). This is because upstream genes have greater pleiotropic effects on a number of pathway end products (Cork & Purugganan, 2004; Pal, Papp, & Lercher, 2006; Rausher et al., 1999). The *Cc‐alpha Gal* is a debranching enzyme catalyzing the postdepositional degree of galactose side branch to determine the mannose to galactose ratio, and thus, it is possible that its position in the GM biosynthesis pathway and occurrence in different isoforms could have played a role in its accelerated rate of accumulating adaptive mutations in *C. canephora*. No positive selection was observed in *Cc‐GMGT* or *Cc‐Man‐S*, which together catalyze the polymerization of core galactomannan structure, despite the latter showing relatively high average *d*_N_/*d*_S_ ratio in some copies, suggesting that it might be under relaxed selective constraint.

In contrast, phylogenetic analysis failed to support nucleotide diversity with most paralogs in species tested forming distinct subclusters. In addition, no obvious orthologous clustering congruent with species phylogeny could be inferred. Similar results have also been observed in other plant cell wall genes (Ahn et al., 2007; Figueiredo, Lashermes, & Aragão, 2011). This may be due to the highly variable and dynamic nature of plant cell walls throughout growth and development process (Campbell & Braam, 1999). Trees revealed that GM pathway genes might have independently evolved or expanded in distinct patterns in different plant species in order to cope with specific biochemical cell wall modification requirements. However, despite the observed high amino acid sequence diversity, the core biochemically active motifs of specific genes were conserved in different plant lineages, allowing them to retain their primary functional properties. Phylogenies of *GMGT* and *UG4E* gene families suggested that they are evolutionary more distant between monocots and dicots which supports previous hypothesis that Nucleotide‐diphospho‐sugar interconversion enzymes (NSEs) families such as *Epimerases* are highly divergent in monocots and dicots plants (Yin, Huang, Gu, Bar‐Peled, & Xu, 2011).

In summary, this study investigated the evolution patterns of five core structural genes of the galactomannan pathway using multi‐copy based analysis and identified significant variation in evolutionary rate among genes. Positive selection detected in the GM branch editing gene *Cc‐alpha Gal* is likely to explain the reason for varying mannose to galactose ratio in coffee seeds. Highly conserved *Cc‐UG4E* and *Cc‐MGT* suggest a strong negative selection, which purge changes that cause deleterious effects on the fitness due to their crucial role in sucrose carbon flux for galactomannan accumulation in coffee seeds. We also observed lineage‐specific gene family expansion for *MGT*, *alpha Gal*, and *Man‐S*, while independent expansion of *GMGT* and *UG4E* gene families in monocots and dicots. Our results provide insights into the evolutionary rate variations in endosperm cell wall storage polysaccharides genes in the diploid *C. canephora*.

## CONFLICT OF INTEREST

The authors declare that they have no competing interests.

## AUTHORS CONTRIBUTION

Mr. Collins Ogutu conceived the idea, designed, and conducted the experiments. Ms. Sylvia Cherono and Ms. Charmaine Ntini helped with the experiments and manuscript revision. Mr. Md. Dulal Ali Mollah, Mr. Lei Zhao, and Mr. Mohammad A. Belal helped with data analysis.

## CONSENT FOR PUBLICATION

Not applicable.

## ETHICS APPROVAL AND CONSENT TO PARTICIPATE

All methods used to collect observational data were noninvasive.

## Data Availability

All DNA sequences have been submitted to the GenBank databases, and accession numbers are provided in the methods section.

## References

[ece36084-bib-0001] Ahn, Y. O. , Zheng, M. , Bevan, D. R. , Esen, A. , Shiu, S.‐H. , Benson, J. , … Poulton, J. E. (2007). Functional genomic analysis of *Arabidopsis thaliana* glycoside hydrolase family 35. Phytochemistry, 68, 1510–1520. 10.1016/j.phytochem.2007.03.021 17466346

[ece36084-bib-0002] Altschul, S. F. , Madden, T. L. , Schaffer, A. A. , Zhang, J. , Zhang, Z. , Miller, W. , & Lipman, D. J. (1997). Gapped BLAST and PSI‐BLAST: A new generation of protein database search programs. Nucleic Acids Research, 25, 3389 10.1093/nar/25.17.3389 9254694 PMC146917

[ece36084-bib-0003] Blanc, G. , & Wolfe, K. H. (2004). Functional divergence of duplicated genes formed by polyploidy during Arabidopsis evolution. The Plant Cell, 16, 1679–1691. 10.1105/tpc.021410 15208398 PMC514153

[ece36084-bib-0004] Braverman, J. M. , Hamilton, M. B. , & Johnson, B. A. (2016). Patterns of substitution rate variation at many nuclear loci in two species trios in the *Brassicaceae* partitioned with ANOVA. Journal of Molecular Evolution, 83, 97–109. 10.1007/s00239-016-9752-x 27592229

[ece36084-bib-0005] Brown, K. E. , & Kelly, J. K. (2018). Antagonistic pleiotropy can maintain fitness variation in annual plants. Journal of Evolutionary Biology, 31, 46–56. 10.1111/jeb.13192 29030895

[ece36084-bib-0006] Buckeridge, M. (2010). Seed cell wall storage polysaccharides: Models to understand cell wall biosynthesis and degradation. Plant Physiology, 154, 1017–1023. 10.1104/pp.110.158642 20855518 PMC2971584

[ece36084-bib-0007] Buckeridge, M. , & Reid, J. (1996). Major cell wall polysaccharides in legume seeds: Structure, catabolism and biological functions. Ciência e Cultura, 48, 153–162.

[ece36084-bib-0008] Campbell, P. , & Braam, J. (1999). Xyloglucan endotransglycosylases: Diversity of genes, enzymes and potential wall‐modifying functions. Trends in Plant Science, 4, 361–366. 10.1016/S1360-1385(99)01468-5 10462769

[ece36084-bib-0009] Charlesworth, D. (2006). Balancing selection and its effects on sequences in nearby genome regions. PLoS Genetics, 2, 64 10.1371/journal.pgen.0020064 PMC1449905 16683038

[ece36084-bib-0010] Chrost, B. , & Schmitz, K. (2000). Purification and characterization of multiple forms of α‐galactosidases in *Cucumis melo* plants. Journal of Plant Physiology, 156, 483–491.

[ece36084-bib-0011] Chu, S. S. , Wang, J. , Cheng, H. , Yang, Q. , & Yu, D. Y. (2014). Evolutionary study of the isoflavonoid pathway based on multiple copies analysis in soybean. BMC Genetics, 15, 76 10.1186/1471-2156-15-76 24962214 PMC4076065

[ece36084-bib-0012] Clotault, J. , Peltier, D. , Soufflet‐Freslon, V. , Briard, M. , & Geoffriau, E. (2012). Differential selection on carotenoid biosynthesis genes as a function of gene position in the metabolic pathway: A study on the carrot and dicots. PLoS ONE, 7, e38724 10.1371/journal.pone.0038724 22737218 PMC3377682

[ece36084-bib-0013] Conklin, P. L. , Norris, S. R. , Wheeler, G. L. , Williams, E. H. , Smirnoff, N. , & Last, R. L. (1999). Genetic evidence for the role of GDP mannose in plant ascorbic acid (vitamin C) biosynthesis. Proceedings of the National Academy of Sciences of the United States of America, 96, 4198–4203. 10.1073/pnas.96.7.4198 10097187 PMC22444

[ece36084-bib-0014] Cork, J. M. , & Purugganan, M. D. (2004). The evolution of molecular genetic pathways and networks. BioEssays, 26, 479–484. 10.1002/bies.20026 15112228

[ece36084-bib-0015] Denoeud, F. , Carretero‐Paulet, L. , Dereeper, A. , Droc, G. , Guyot, R. , Pietrella, M. , … Lashermes, P. (2014). The coffee genome provides insight into the convergent evolution of caffeine biosynthesis. Science, 345, 1181–1184. 10.1126/science.1255274 25190796

[ece36084-bib-0016] Dey, P. M. , & Del Campillo, E. (1984). Biochemistry of multiple forms of glycosidases in plants. Advances in Enzymology and Related Areas of Molecular Biology, 56, 141–249. 6320603 10.1002/9780470123027.ch3

[ece36084-bib-0017] Dhugga, K. , Barreiro, R. , Whitten, B. , Stecca, K. , Hazebroek, J. , Randhawa, G. S. , … Anderson, P. (2004). Guar seed betamannan synthase is a member of the cellulose synthase super gene family. Science, 303, 363–366. 14726589 10.1126/science.1090908

[ece36084-bib-0018] Edwards, M. , Scott, C. , Gidley, M. J. , & Reid, J. S. G. (1992). Control of mannose galactose ratio during galactomannan formation in developing legume seeds. Planta, 187, 67–74. 10.1007/BF00201625 24177968

[ece36084-bib-0019] Figueiredo, S. A. , Lashermes, P. , & Aragão, F. J. (2011). Molecular characterization and functional analysis of the b‐galactosidase gene during *Coffea arabica* (L.) fruit development. Journal of Experimental Botany, 62, 2691–2703. 21239378 10.1093/jxb/erq440

[ece36084-bib-0020] Gaut, B. , Yang, L. , Takuno, S. , & Eguiarte, L. E. (2011). The patterns and causes of variation in plant nucleotide substitution rates. Annual Review of Ecology, Evolution, and Systematics, 42, 245–266. 10.1146/annurev-ecolsys-102710-145119

[ece36084-bib-0021] Gish, W. , & States, D. J. (1993). Identification of protein coding regions by database similarity search. Nature Genetics, 3, 266 10.1038/ng0393-266 8485583

[ece36084-bib-0022] Greenberg, A. J. , Stockwell, S. R. , & Clark, A. G. (2008). Evolutionary constraint and adaptation in the metabolic network of Drosophila. Molecular Biology and Evolution, 25, 2537–2546. 10.1093/molbev/msn205 18799713 PMC2721553

[ece36084-bib-0023] Gruppen, H. , Kormelink, F. , & Voragen, A. (1993). Water‐unextractable cell wall material from wheat flour. 3. A structural model for arabinoxylans. Journal of Cereal Science., 18, 111–128. 10.1006/jcrs.1993.1040

[ece36084-bib-0024] Gu, Z. , Cavalcanti, A. , Chen, F. , Bouman, P. , & Li, W. (2002). Extent of gene duplication in the genomes of Drosophila, nematode, and yeast. Molecular Biology and Evolution, 19, 256–262. 10.1093/oxfordjournals.molbev.a004079 11861885

[ece36084-bib-0025] Guevara, D. R. , El‐Kereamy, A. , Yaish, M. W. , Mei‐Bi, Y. , & Rothstein, S. J. (2014). Functional characterization of the rice UDP‐glucose 4‐epimerase 1, OsUGE1: A potential role in cell wall carbohydrate partitioning during limiting nitrogen conditions. PLoS ONE, 9, e96158. 24788752 10.1371/journal.pone.0096158 PMC4006880

[ece36084-bib-0026] Hall, T. A. (1999). BioEdit: A user‐friendly biological sequence alignment editor and analysis program for Windows 95/98/NT. Nucleic Acids Symposium Series, 41, 95–98.

[ece36084-bib-0027] Horbowicz, M. , & Obendorf, R. L. (1994). Seed desiccation tolerance and storability: Dependence on flatulence‐producing oligosaccharides and cyclitols—Review and survey. Seed Science Research, 4, 385–405. 10.1017/S0960258500002440

[ece36084-bib-0028] Huang, Y. , Niu, B. , Gao, Y. , Fu, L. , & Li, W. (2010). CD‐HIT Suite: A web server for clustering and comparing biological sequences. Bioinformatics, 26, 680–682. 10.1093/bioinformatics/btq003 20053844 PMC2828112

[ece36084-bib-0029] Jeffares, D. C. , Tomiczek, B. , Sojo, V. , & dos Reis, M. (2015). A beginners guide to estimating the nonsynonymous to synonymous rate ratio of all protein-coding genes in a genome. Methods in Molecular Biology., 1201, 65–90. 25388108 10.1007/978-1-4939-1438-8_4

[ece36084-bib-0030] Jeffrey, L. (2002). Mechanisms and rates of genome expansion and contraction in flowering plants. Genetica, 115, 29–36. 12188046 10.1023/a:1016015913350

[ece36084-bib-0031] Joersbo, M. , Marcussen, J. , & Brunstedt, J. (2001). In vivo modification of the cell wall polysaccharide galactomannan of guar transformed with an alpha‐galactosidase gene cloned from senna. Molecular Breeding, 7, 211–219.

[ece36084-bib-0032] Joët, T. , Laffargue, A. , Salmona, J. , Doulbeau, S. , Descroix, F. , Bertrand, B. , … Dussert, S. (2014). Regulation of galactomannan biosynthesis in coffee seeds. Journal of Experimental Botany, 65, 323–337. 10.1093/jxb/ert380 24203356

[ece36084-bib-0033] Kazutaka, K. , & Daron, M. S. (2013). MAFFT multiple sequence alignment software version 7: Improvements in performance and usability. Molecular Biology and Evolution, 30, 772–780. 10.1093/molbev/mst010 23329690 PMC3603318

[ece36084-bib-0034] Kimura, M. (1977). Preponderance of synonymous changes as evidence for the neutral theory of molecular evolution. Nature, 267, 275–276. 10.1038/267275a0 865622

[ece36084-bib-0035] Kryazhimskiy, S. , & Plotkin, J. B. (2008). The population genetics of dN/dS. PLoS Genetics, 4(12), e1000304 10.1371/journal.pgen.1000304 19081788 PMC2596312

[ece36084-bib-0036] Lavin, M. , Herendeen, P. S. , & Wojciechowski, M. F. (2005). Evolutionary rates analysis of Leguminosae implicates a rapid diversification of lineages during the tertiary. Systematic Biology, 54, 575–594. 10.1080/10635150590947131 16085576

[ece36084-bib-0037] Librado, P. , & Rozas, J. (2009). DnaSP v5: A software for comprehensive analysis of DNA polymorphism data. Bioinformatics, 25, 1451–1452. 10.1093/bioinformatics/btp187 19346325

[ece36084-bib-0038] Livingstone, K. , & Anderson, S. (2009). Patterns of variation in the evolution of carotenoid biosynthetic pathway enzymes of higher plants. Journal of Heredity, 100, 754–761. 10.1093/jhered/esp026 19520763

[ece36084-bib-0039] Lu, Y. , & Rausher, M. D. (2003). Evolutionary rate variation in anthocyanin pathway genes. Molecular Biology and Evolution, 20, 1844–1853. 10.1093/molbev/msg197 12885963

[ece36084-bib-0040] Lynch, M. , & Conery, J. S. (2000). The evolutionary fate and consequences of duplicate genes. Science, 290, 1151–1155. 10.1126/science.290.5494.1151 11073452

[ece36084-bib-0041] Ma, Y. , Wang, J. , Zhong, Y. , Geng, F. , Cramer, G. R. , & Cheng, Z. M. (2015). Subfunctionalization of cation/proton antiporter 1 genes in grapevine in response to salt stress in different organs. Horticulture Research, 2, 15031 10.1038/hortres.2015.31 26504576 PMC4591679

[ece36084-bib-0042] Maria, A. , Joseph, P. , & Ziheng, Y. (2001). Accuracy and power of the likelihood ratio test in detecting adaptive molecular evolution. Molecular Biology and Evolution, 18, 1585–1592. 11470850 10.1093/oxfordjournals.molbev.a003945

[ece36084-bib-0043] Nielsen, R. , & Yang, Z. (2003). Estimating the distribution of selection coefficients from phylogenetic data with applications to mitochondrial and viral DNA. Molecular Biology and Evolution, 20, 1231–1239. 10.1093/molbev/msg147 12777508

[ece36084-bib-0044] Ogutu, C. , Fang, T. , Yan, L. , Wang, L. , Huang, L. , Wang, X. , … Han, Y. (2016). Characterization and utilization of microsatellites in the *Coffea canephora* genome to assess genetic association between wild species in Kenya and cultivated coffee. Tree Genetics and Genomes, 12, 54 10.1007/s11295-016-1014-y

[ece36084-bib-0045] Pal, C. , Papp, B. , & Lercher, M. J. (2006). An integrated view of protein evolution. Nature Reviews Genetics, 7, 337–348. 10.1038/nrg1838 16619049

[ece36084-bib-0046] Panchy, N. , Lehti‐Shiu, M. , & Shiu, S. H. (2016). Evolution of gene duplication in plants. Plant Physiology, 171, 2294–2316. 10.1104/pp.16.00523 27288366 PMC4972278

[ece36084-bib-0047] Peden, J. F. (1999). CodonW: Analysis of codon usage. PhD thesis, University of Nottingham, UK.

[ece36084-bib-0048] Pierce, V. , & Crawford, D. (1997). Phylogenetic analysis of glycolytic enzyme expression. Science, 276, 256–259. 10.1126/science.276.5310.256 9092475

[ece36084-bib-0049] Polhill, R. M. , Raven, P. H. , & Stirto, C. H. (1981). Evolution and Systematics of the Leguminosae In Polhill R. M., & Raven P. H. (Eds.), Advances in Legume Systematics (pp. 1–26). Kew: Part I, Royal Botanic Garden.

[ece36084-bib-0050] Popescu, C. E. , Borza, T. , Bielawski, J. P. , & Lee, R. W. (2006). Evolutionary rates and expression level in Chlamydomonas. Genetics, 172, 1567–1576. 10.1534/genetics.105.047399 16361241 PMC1456299

[ece36084-bib-0051] Pré, M. , Caillet, V. , Sobilo, J. , & McCarthy, J. (2008). Characterization and expression analysis of genes directing galactomannan synthesis in coffee. Annals of Botany, 102, 207–220. 10.1093/aob/mcn076 18562467 PMC2712370

[ece36084-bib-0052] Purugganan, M. , & Wessler, S. (1994). Molecular evolution of the plant R regulatory gene family. Genetics, 138, 849–854. 7851779 10.1093/genetics/138.3.849 PMC1206232

[ece36084-bib-0053] Ramsay, H. , Rieseberg, L. H. , & Ritland, K. (2009). The correlation of evolutionary rate with pathway position in plant terpenoid biosynthesis. Molecular Biology and Evolution, 26, 1045–1053. 10.1093/molbev/msp021 19188263

[ece36084-bib-0054] Rausher, M. , Lu, Y. , & Meyer, K. (2008). Variation in constraint versus positive selection as an explanation for evolutionary rate variation among anthocyanin genes. Journal of Molecular Evolution, 67, 137–144. 10.1007/s00239-008-9105-5 18654810

[ece36084-bib-0055] Rausher, M. , Miller, R. , & Tiffin, P. (1999). Patterns of evolutionary rate variation among genes of the anthocyanin biosynthetic pathway. Molecular Biology and Evolution, 16, 266–274. 10.1093/oxfordjournals.molbev.a026108 10028292

[ece36084-bib-0056] Redgwell, R. , & Fischer, M. (2006). Coffee carbohydrates. Brazilian Journal of Plant Physiology, 18, 165–174. 10.1590/S1677-04202006000100012

[ece36084-bib-0057] Reid, J. , Edwards, M. , Dickson, C. , Scott, C. , & Gidley, M. (2003). Tobacco transgenic lines that Express fenugreek galactomannan galactosyltransferase constitutively have structurally altered galactomannans in their seed endosperm cell walls. Plant Physiology, 131, 1487–1495. 10.1104/pp.102.016840 12644698 PMC166908

[ece36084-bib-0058] Schmid, K. J. , Ramos‐Onsins, S. , Ringys‐Beckstein, H. , Weisshaar, B. , & Mitchell‐Olds, T. (2005). A multilocus sequence survey in *Arabidopsis thaliana* reveals a genome‐wide departure from a neutral model of DNA sequence polymorphism. Genetics, 169, 1601–1615. 15654111 10.1534/genetics.104.033795 PMC1449538

[ece36084-bib-0059] Sharp, P. (1991). Determinants of DNA sequence divergence between *Escherichia coli* and *Salmonella typhimurium*: Codon usage, map position, and concerted evolution. Journal of Molecular Evolution, 33, 23–33. 10.1007/BF02100192 1909371

[ece36084-bib-0060] Sharp, P. , Tuohy, T. , & Mosurski, K. (1986). Codon usage in yeast: Cluster analysis clearly differentiates highly and lowly expressed genes. Nucleic Acids Research, 14, 5125–5143. 10.1093/nar/14.13.5125 3526280 PMC311530

[ece36084-bib-0061] Siol, M. , Wright, S. I. , & Barrett, S. C. (2010). The population genomics of plant adaptation. New Phytologist, 188, 313–332. 10.1111/j.1469-8137.2010.03401.x 20696011

[ece36084-bib-0062] Sullivan, M. J. , Petty, N. K. , & Beatson, S. A. (2011). Easyfig: A genome comparison visualizer. Bioinformatics, 27, 1009–1010. 10.1093/bioinformatics/btr039 21278367 PMC3065679

[ece36084-bib-0063] Swanson, W. J. , Nielsen, R. , & Yang, Q. (2003). Pervasive adaptive evolution in mammalian fertilization proteins. Molecular Biology and Evolution, 20, 18–20. 10.1093/oxfordjournals.molbev.a004233 12519901

[ece36084-bib-0064] Tamura, K. , Stecher, G. , Peterson, D. , Filipski, A. , & Kumar, S. (2013). MEGA6: Molecular evolutionary genetics analysis version 6.0. Molecular Biology and Evolution, 30, 2725–2729. 10.1093/molbev/mst197 24132122 PMC3840312

[ece36084-bib-0065] Thompson, J. D. , Higgins, D. G. , & Gibson, T. J. (1994). CLUSTAL W: Improving the sensitivity of progressive multiple sequence alignment through sequence weighting, position‐specific gap penalties and weight matrix choice. Nucleic Acids Research, 22, 4673–4680. 7984417 10.1093/nar/22.22.4673 PMC308517

[ece36084-bib-0066] Toole, G. A. , Le Gall, G. , Colquhoun, I. J. , Drea, S. , Opanowicz, M. , Bedő, Z. , … Mills, E. (2012). Spectroscopic analysis of diversity in the spatial distribution of arabinoxylan structures in endosperm cell walls of cereal species in the HEALTHGRAIN diversity collection. Journal of Cereal Science, 56, 134–141. 10.1016/j.jcs.2012.02.016

[ece36084-bib-0067] Tucker, P. , & Lundrigan, B. (1993). Rapid evolution of the sex‐determining loci in Old World mice and rats. Nature, 364, 715–717. 8355784 10.1038/364715a0

[ece36084-bib-0068] Vitkup, D. , Kharchenko, P. , & Wagner, A. (2006). Influence of metabolic network structure and function on enzyme evolution. Genome Biology, 7, R39. 16684370 10.1186/gb-2006-7-5-r39 PMC1779518

[ece36084-bib-0069] Wright, F. (1990). The “effective number of codons” used in a gene. Gene, 87, 23–29. 10.1016/0378-1119(90)90491-9 2110097

[ece36084-bib-0070] Wright, S. I. , & Gaut, B. S. (2005). Molecular population genetics and the search for adaptive evolution in plants. Molecular Biology and Evolution, 22, 506–519. 10.1093/molbev/msi035 15525701

[ece36084-bib-0071] Yan, L. , Ogutu, C. , Huang, L. , Wang, X. , Zhou, H. , Lv, Y. , … Han, Y. (2019). Genetic diversity and population structure of coffee germplasm collections in China revealed by ISSR markers. Plant Molecular Biology Reporter, 37, 204–213. 10.1007/s11105-019-01148-3

[ece36084-bib-0072] Yang, Y. H. , Zhang, F. M. , & Ge, S. (2009). Evolutionary rate patterns of the gibberellin pathway genes. BMC Evolutionary Biology, 9, 206 10.1186/1471-2148-9-206 19689796 PMC2794029

[ece36084-bib-0073] Yang, Z. (1997). PAML: A program package for phylogenetic analysis by maximum likelihood. Computer Applications in the Biosciences, 13, 555–556. 10.1093/bioinformatics/13.5.555 9367129

[ece36084-bib-0074] Yang, Z. , & Nielsen, R. (2000). Estimating synonymous and nonsynonymous substitution rates under realistic evolutionary models. Molecular Biology and Evolution, 17, 32–43. 10.1093/oxfordjournals.molbev.a026236 10666704

[ece36084-bib-0075] Yin, Y. , Huang, J. , Gu, X. , Bar‐Peled, M. , & Xu, Y. (2011). Evolution of plant nucleotide sugar interconversion enzymes. PLoS ONE, 6, e27995 10.1371/journal.pone.0027995 22125650 PMC3220709

