# Supplementary material for: Evolutionary rate variation among genes involved in galactomannan biosynthesis in Coffea canephora
Source: Ecol Evol. 2020 Feb 11;10(5):2559–69. doi: 10.1002/ece3.6084 (PMC7069334; doi:10.1002/ece3.6084)
Supplement: Supplementary file 1 [file ECE3-10-2559-s001.zip › ece36084-sup-0004-Supinfo.docx]

Table S1. List of primers used in this study

| **gene** | **locus** | **Forward primer 5’-3’** | **Reverse primer 5’-3’** | **Tm (°C)** |
| --- | --- | --- | --- | --- |
| *Cc-ManS* | Cc06_g04240 | ATGAGAAACTCAGTTTTTCTAG | TTAAGTAGGGACAATTGTTCCA | 56 |
|  | Cc01_g05870 | ATGGCCCCAAGCTCTGTGGTG | TTAGCTTACCTGCTCTCCGA | 61 |
|  | Cc06_g05120 | ATGGATAGGCTTCCATCCGC | TTATGAGTTGGGAACAAAAGTTC | 55 |
|  | Cc08_g06540 | ATGGCTCCAATATTGGAT | TCAGCTCATTTGCTCCCCAAT | 53 |
|  | Cc10_g07720 | ATGGCACCATCATTTGACTGGT | CTAGTCAACCTGCTCCCCTATC | 58 |
| *Cc-GMGT* | Cc07_g07220 | ATGACCAGAATCAAGGTTCACACC | CTACACCAATTCTTCCTTCTCGTCG | 60 |
|  | Cc07_g07210 | ATGCCTAAGCACAACAGCCTCCTCC | CTAGGAAGCGCCTTCAACAAGGTCC | 55 |
|  | Cc00_g25840 | ATGGGCCAAGAGAGCAATTTTACAC | TCAACCTGCTCCTCTTTTAGAGTGA | 60 |
|  | Cc03_g01930 | ATGCTGGAAAGATGCTTGAGCC | CTAAGAAGCAGAAACCTTAACAGCT | 57 |
| *Cc-alpha Gal* | Cc02_g05490 | ATGGCGCCTGTACTTATAACAATCA | TTACAGAATCTGAGAGTTAGTAG | 58 |
|  | Cc11_g15950 | ATGCAGATTTTAGCTCTGATTG | TTATAAACTTGCCTCCAT | 50 |
|  | Cc04_g14280 | ATGGTGAAGTCTCCAGGAACCGAGG | TCACTGTGGGGTTAGGACATACATT | 51 |
|  | Cc11_g00330 | ATGGAGGACAGGAAGAAGCCATC | TCAAGACCTATCTGGGGTGAAGATA | 56 |
| *Cc-UG4E* | Cc11_g04810 | ATGTTAGCGAGTCATATGGCAAGTG | TTAGGTAATGGTTAAAGTAACAGAG | 55 |
|  | Cc07_g03170 | ATGTCAAGACTTTCATTCTTG | CTATTCGTGTTTGGTCTTG | 60 |
|  | Cc00_g06000 | ATGTTAGCGAGTCATATGGCAAGT | TTAGGTAATGGTTAAAGTAACAGAG | 57 |
| *Cc-MGT* | Cc05_g00320 | ATGAAGGCACTTATTCTTGTTGGAG | TCACATAACAATCTCTGACTTCAAG | 54 |
|  | Cc02_g07370 | ATGAAGGCACTTATTCTTGTTGGAG | TCACATAACAATCTCTGGCTTCAAG | 53 |

Table S2. List of *C. canephora* accessions used in this study

| No. | Accession | sample | Origin | No. | Accession | Sample | Origin |
| --- | --- | --- | --- | --- | --- | --- | --- |
| 1 | 24-1 | seeds | Asia | 13 | LG3-1 | seeds | Europe |
| 2 | Xwang-8 | seeds | Asia | 14 | Xwang-4 | seeds | Asia |
| 3 | 24-1a | seeds | Asia | 15 | Lg-01 | seeds | Europe |
| 4 | CC-6 | seeds | Africa* | 16 | CC-18 | seeds | Africa |
| 5 | cc-1 | seeds | Africa* | 17 | CC-14 | seeds | Africa* |
| 6 | 26-cc8 | seeds | Asia | 18 | 26-cc4 | seeds | Asia |
| 7 | Sg-1 | seeds | Asia | 19 | Xwang-M | seeds | Asia |
| 8 | Sg-5 | seeds | S. America | 20 | 24-1M | seeds | Asia |
| 9 | Lg-8 | seeds | S. America | 21 | 26-cc9 | seeds | Europe |
| 10 | Sg-5 | seeds | Africa* | 22 | Bg-10 | seeds | Europe |
| 11 | Lg-M | seeds | Africa | 23 | Bg-M2 | seeds | Europe |
| 12 | cc-4 | seeds | Africa* |  |  |  |  |

*Represents wild accessions used in this study.

**
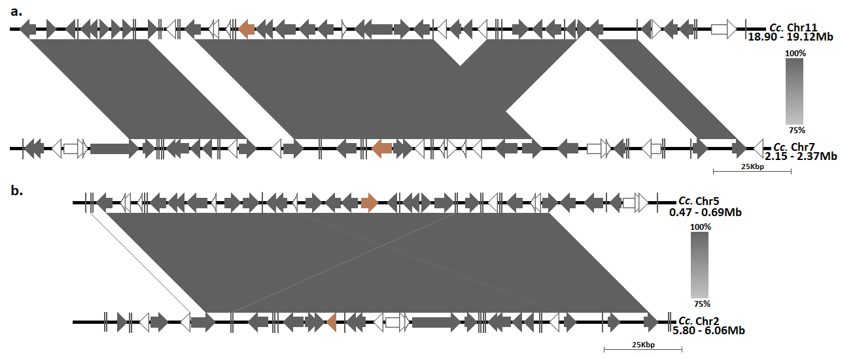
**

Fig S1. Synteny analysis of tandem chromosomal regions for *Cc*-UG4E (a) and *Cc*-MGT (b) genes in *C. canephora* genome showing extensive syntenic alignments (> 60%) of the two chromosome segments. Arrow heads represent different segment features, and target gene position is shown in dark brown color. Color intensity of grey diagonal blocks represents rate of conserved sequence sites on chromosomes.


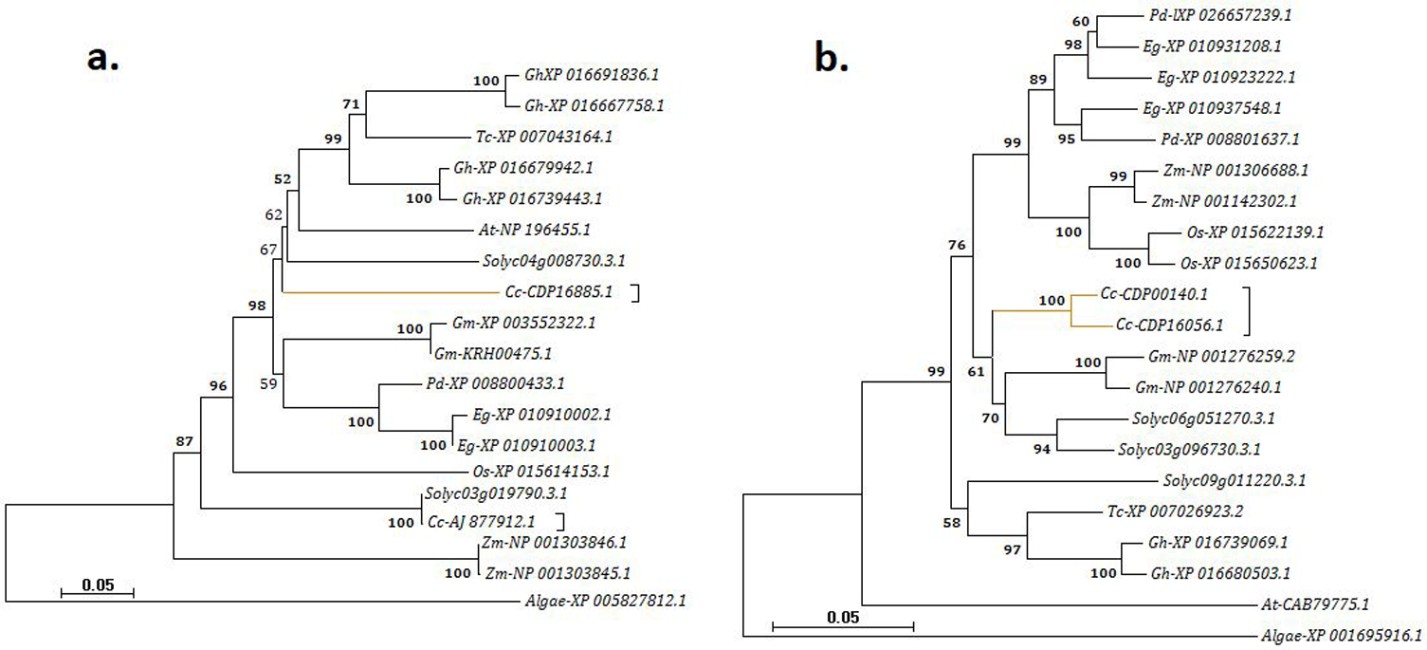


Fig S2. Consensus phylogenetic trees of (a) *alpha Gal* and (b) *MGT* gene family members from different plant lineages. Italicized abbreviations represents species name: *Tc-* *Theobroma cocoa*, *Gh-* *Gossypium hirsutum*, *At- Arabidopsis thaliana*, *Cc- Coffea canephora*, *Gm- Glycine max*, *Eg- Elaeis guineensis*, *Pd- Phoenix dactylifera*, *Os- Oryza sativa*, *Zm- Zea mays, Solyc- Solanum lycopersicum*. Gene accession numbers are shown in-front of species name and bootstrap values indicating confidence levels ≥50 for clustering are shown at the nodes. Tree branches for *C. canephora* copies are highlighted in orange color.


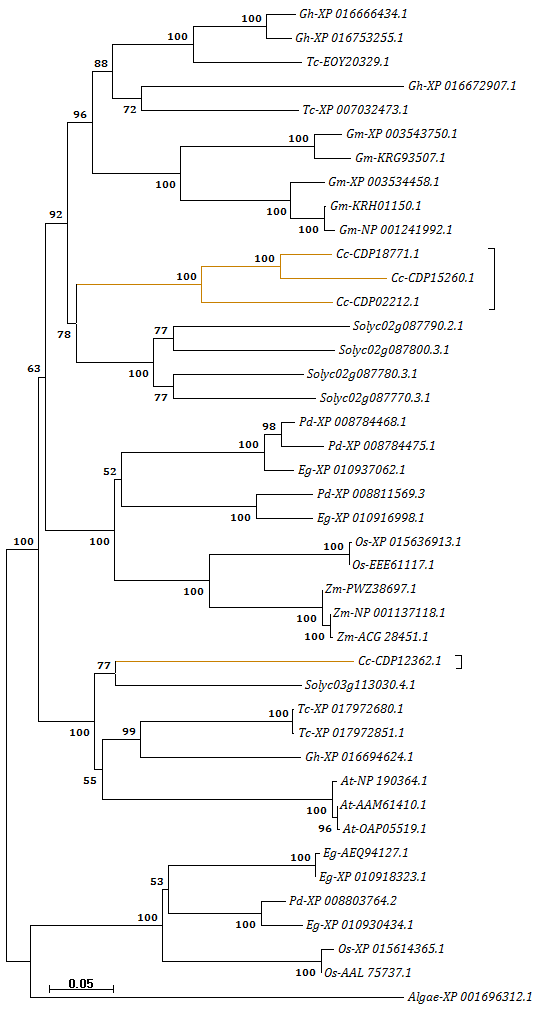


Fig S3. Consensus phylogenetic trees of *UG4E* gene family members from different plant lineages. Italicized abbreviations represents species name: *Tc-* *Theobroma cocoa*, *Gh-* *Gossypium hirsutum*, *At- Arabidopsis thaliana*, *Cc- Coffea canephora*, *Gm- Glycine max*, *Eg- Elaeis guineensis*, *Pd- Phoenix dactylifera*, *Os- Oryza sativa*, *Zm- Zea mays, Solyc- Solanum lycopersicum*. Gene accession numbers are shown in-front of species name and bootstrap values indicating confidence levels ≥50 for clustering are shown at the nodes. Tree branches for *C. canephora* copies are highlighted in orange color.
