# Supplementary figures and images for: Evolutionary rate variation among genes involved in galactomannan biosynthesis in Coffea canephora
Source: Ecol Evol. 2020 Feb 11;10(5):2559–69. doi: 10.1002/ece3.6084 (PMC7069334; doi:10.1002/ece3.6084)

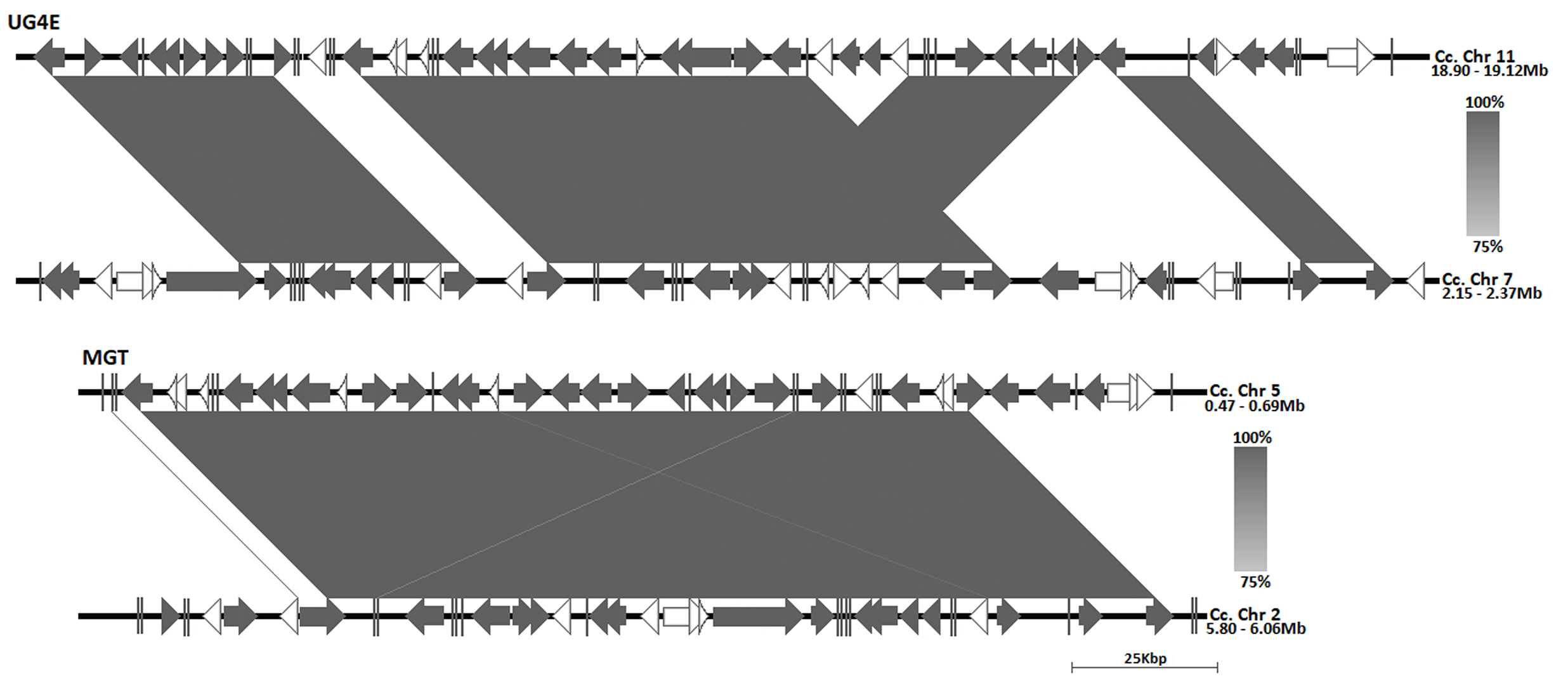

Supplement: Supplementary file 1 [file ECE3-10-2559-s001.zip › ece36084-sup-0001-FigS1.tif]

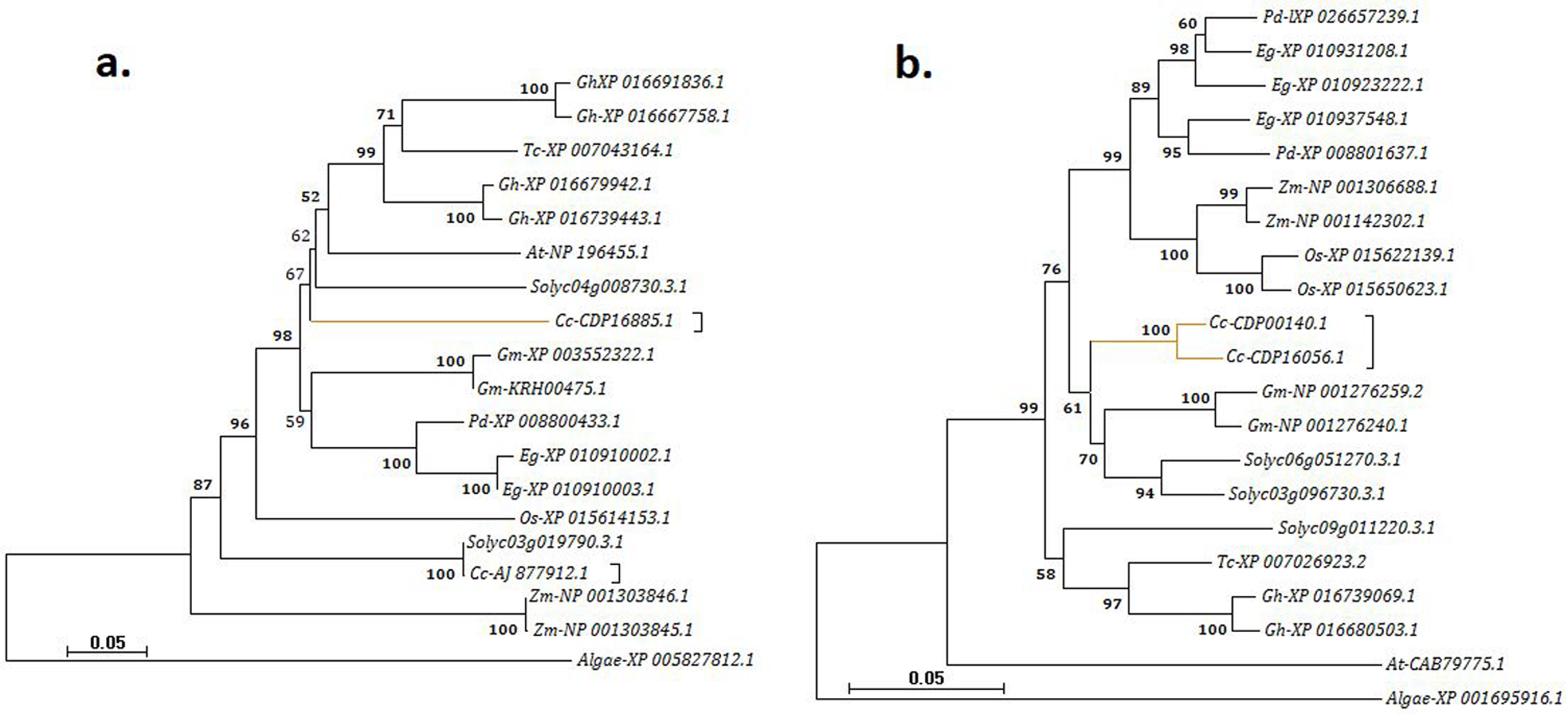

Supplement: Supplementary file 1 [file ECE3-10-2559-s001.zip › ece36084-sup-0002-FigS2.tif]

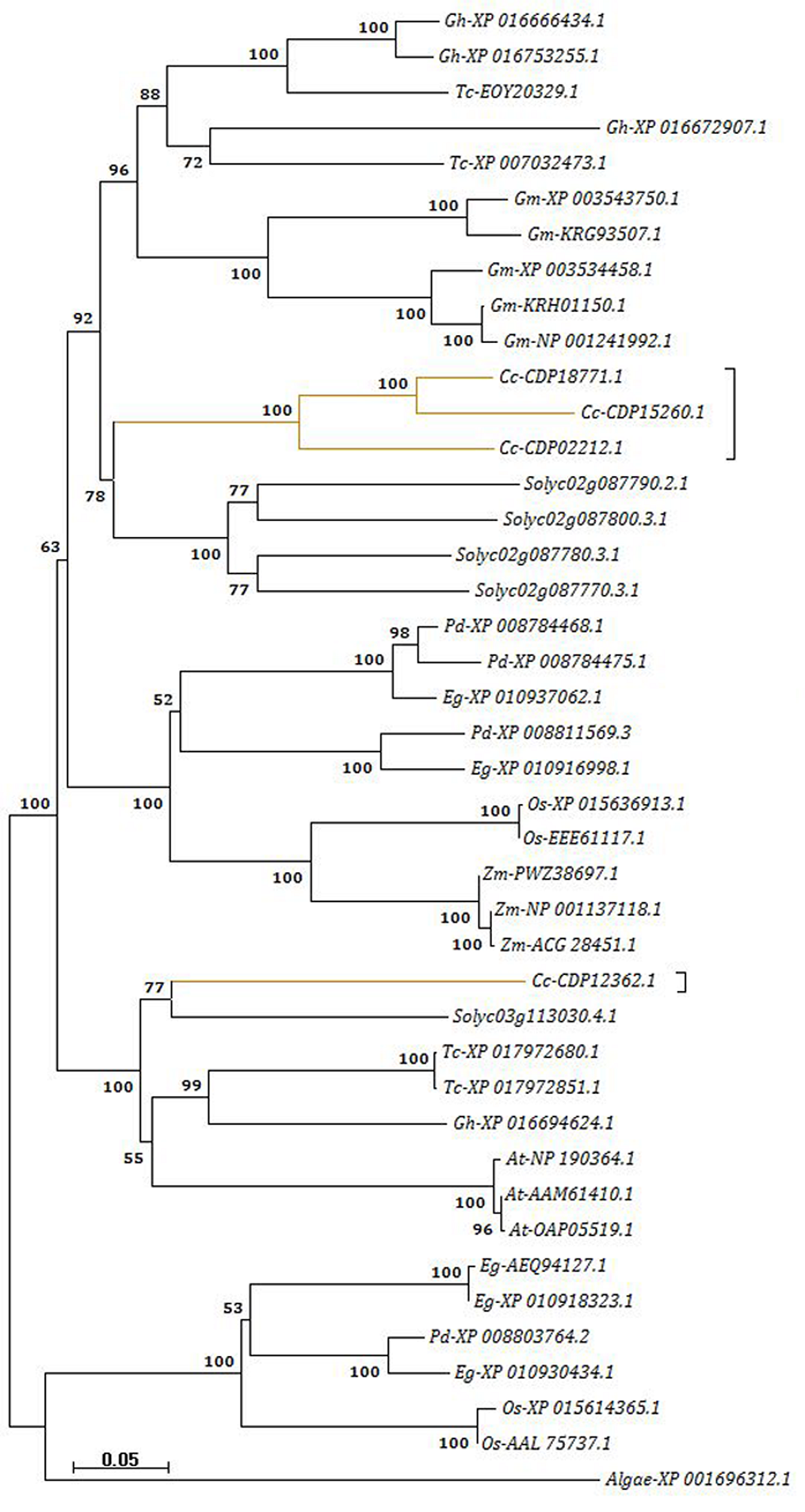

Supplement: Supplementary file 1 [file ECE3-10-2559-s001.zip › ece36084-sup-0003-FigS3.tif]
